# Preparation of Fast Dissolving Films for Oral Dosage from Natural Polysaccharides

**DOI:** 10.3390/ma3084291

**Published:** 2010-08-09

**Authors:** Yoshifumi Murata, Takashi Isobe, Kyoko Kofuji, Norihisa Nishida, Ryosei Kamaguchi

**Affiliations:** 1 Faculty of Pharmaceutical Science, Hokuriku University, Ho-3, Kanagawa-machi, Kanazawa 920-1181, Japan; 2 Morishita Jintan Co. Osaka Technocenter, 2–11–1, Tudayamate, Hirakata, Osaka 573–0128, Japan

**Keywords:** fast dissolving film, natural polysaccharides, dexamethasone, pilocarpine, lactoferin

## Abstract

Fast-dissolving films (FDFs) were prepared from natural polysaccharides, such as pullulan, without heating, controlling the pH, or adding other materials. The release profiles of model drugs from the films were investigated. In the absence of a drug, the casting method and subsequent evaporation of the solvent resulted in the polysaccharide forming a circular film. The presence of drugs (both their type and concentration) affected film formation. The thickness of the film was controllable by adjusting the concentration of the polysaccharide, and regular unevenness was observed on the surface of 2% pullulan film. All films prepared with polysaccharides readily swelled in dissolution medium, released the incorporated compound, and subsequently disintegrated. The release of dexamethasone from the films was complete after 15 min, although this release rate was slightly slower than that of pilocarpine or lidocaine. Therefore, FDFs prepared from polysaccharides could be promising candidates as oral dosage forms containing drugs, and would be expected to show drug dissolution in the oral cavity.

## 1. Introduction

Numerous oral disintegration (OD) dosage forms have been developed, with tablets taken with water being the most widely utilized. OD tablets absorb saliva and immediately disintegrate in the oral cavity. After disintegration, the drug and the insoluble components, such as the disintegrated material incorporated in the OD tablet, remain on or around the tongue. This dosage form, however, may not be easy to swallow, so the development of new forms for patients who have difficulty swallowing regular tablets is desirable. Recently, fast-dissolving films (FDFs) have attracted interest as an excellent dosage form, not only for oral care, but also for patients with aphagia or dysphagia [1,2]. FDFs are generally prepared from a polymer base and a plasticizer such as sorbitol, fatty acid or polyvinyl alcohol [3,4,5].

Natural polysaccharides and polypeptides have been studied as materials for food or dosage forms [6,7,8]. These polymers are also utilized for film preparation; for example, agar and gelatin are popular because of their safety and ready supply. When a film is prepared with polysaccharides or polypeptides, the aqueous medium containing the material is heated, the polymer dissolves in the solvent, and then the resultant solution is cooled [9,10]. Chitosan is a desirable material for film formation, but the pH of the solvent must be lowered to effect complete dissolution because chitosan is a cationic polysaccharide obtained by the deacetylation of chitin [11,12]. When a film dosage form is prepared with such polysaccharides, incorporated active compounds such as drugs are exposed in the environment. Thus, the film affects the stability of the incorporated compound. Ideally, a FDF should immediately disintegrate and release its components into the oral cavity. It is therefore preferable to prepare FDFs without plasticizer because these additives may affect the normal microflora of the oral cavity, such as *Streptococcus mutans* [13]. Furthermore, the compounds incorporated in the FDF should be carefully selected because the drug loading capacity of films is typically very low.

In this study, the preparation of FDFs from natural polysaccharides without heating, controlling the pH or adding other materials was investigated. Pullulan (PUL), a neutral polysaccharide composed of α-D-maltotriose, and acidic polysaccharides, such as sodium alginate (ALG) and sodium chondroitin sulfate (CHS), were selected for FDF preparation. A polysaccharide produced by *Bifidobacterium longum* JBL05 (BSP) was also investigated as a new material for the preparation of FDF [14]. Two water-soluble drugs (pilocarpine hydrochloride, PC and lidocaine hydrochloride, LD), a slightly soluble drug (dexamethasone, DM), and a polymer compound (lactoferin, LF) were employed as model compounds because these compounds were expected to show action following dissolution in the oral cavity. The release profile of each compound from the film dosage form was investigated.

## 2. Results and Discussion

To form films by the casting method, a polysaccharide solution containing a drug is prepared without heating and poured into a Petri dish. Therefore, the viscosity of the solution is an important factor for casting. The viscosity of each polysaccharide solution is shown in Table 1. As 2–8% PUL and 2% CHS solutions showed low viscosity, they could be readily cast. On the other hand, 2% high molecular weight ALG (H-ALG) solution could not be cast because of its high viscosity (180 mPa∙s) though both 1.5% H-ALG and 2% low molecular ALG (L-ALG) could be cast. 0.5% BSP also had sufficiently low viscosity for casting.

**Figure 1:**
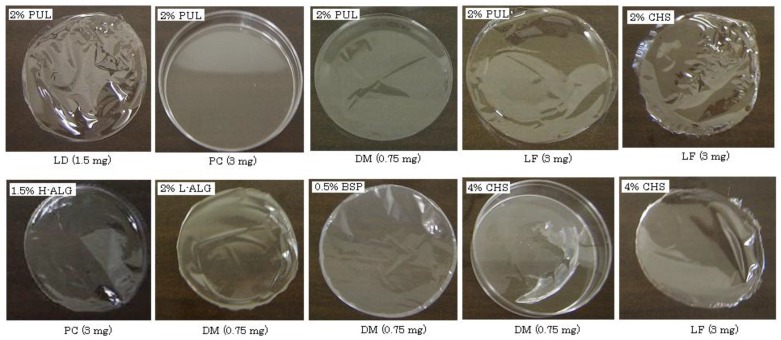
Pictures of FDFs prepared with polysaccharides containing various model compounds.

In the absence of drugs, 2–8% PUL, 1–3% L-ALG, 2% CHS or 0.5% BSP formed a circular film about 5 cm in diameter after evaporation of the solvent. In the case of PUL, a rigid film was obtained and the thickness increased according to the concentration of PUL, as shown in Table 2. L-ALG, CHS and BSP formed soft films, and a thin film (<10-μm thickness) was obtained with 0.5% BSP. Films were also formed when model compounds were present in each polysaccharide solution. For example, in the case of 2% PUL, films were obtained in the presence of LD (1.5 mg), DM (0.75 mg) or LF (3 mg) but not PC (3 mg), as shown in Figure 1. With 4% PUL, all model compounds could be incorporated into the films. On the other hand, 4% CHS did not form a circular film when it contained LD, PC or DM. Thus, the polysaccharides, other than CHS, were able to incorporate each compound used in this study regardless of the compound’s water-solubility or molecular weight. High loading of model compounds interfered with film formation, and 2% PUL, 2% L-ALG and 0.5% BSP could not form circular films in the presence of 15 mg LD. Concentrations above 4% PUL or 3% L-ALG were required to prepare films incorporating 15 mg LD. These results indicate that FDF may be impractical for oral drug administration if large amounts of drug (e.g., 100 mg) are necessary for pharmacological action.

**Table 1:** Viscosities of polysaccharide solutions at 20 °C.

	Viscosity (mPa・s)
2% PUL	< 10
4% PUL	12
8% PUL	48
1.5% H-ALG	130
2% L-ALG	105
2% CHS	< 10
4% CHS	< 10
0.5% BSP	32

**Table 2:** Thicknesses of films prepared with various polysaccharides.

	Thickness (μm, mean ± SD)
2% PUL	23 ± 1
4% PUL	39 ± 4
8% PUL	69 ± 4
1.5% H-ALG	15 ± 1
2% L-ALG	23 ± 1
2% CHS	19 ± 2
4% CHS	29 ± 1
0.5% BSP	< 10

**Figure 2:**
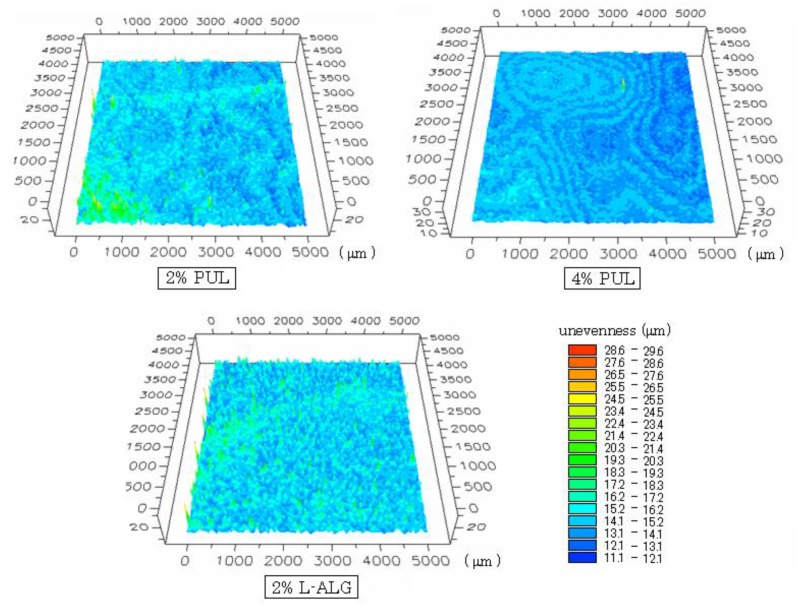
Surface structure of FDFs observed using a three-dimensional profilemeter. Figures show the center of the FDF (square area; 5 × 5 mm). Each color represents the depth of unevenness.

The surface shapes of films (containing 1.5 mg DM) observed using a three-dimensional profilemeter are shown in Figure 2. Although the surface of the 2% PUL film was smooth to the naked eye, regular unevenness was observed on the surface, and the variation in unevenness was 7–16 μm. The range of variation was 12–15 μm for 4% PUL film and 7–16 μm for film prepared with 2% L-ALG.

**Figure 3:**
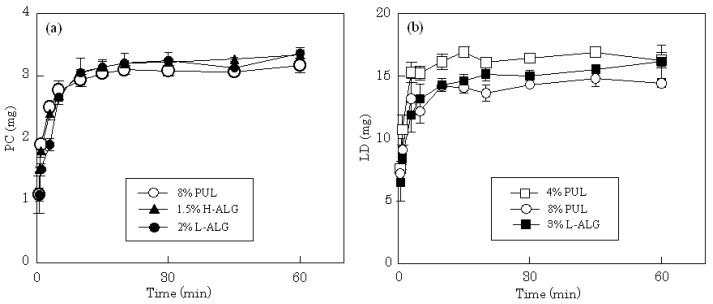
Release profiles of PC (a) and LD (b) from FDFs in physiological saline.

FDFs are expected to dissolve immediately in saliva, which is secreted from salivary glands at 0.5–0.6 L/day (1.5–2.0 mL/min when stimulated) [15]. In this study, all the films readily swelled in 10 mL of dissolution medium, released the incorporated compound and subsequently disintegrated. The release profiles of LD and PC from different film dosage forms in physiological saline are shown in Figure 3. The total amount of PC incorporated in each film, 3 mg, was released after 5-10 min from films prepared with 8% PUL. The same drug release rate was obtained on 4% PUL film. The release profile was attributed to the high solubility of PC in water; rapid release rates were also observed from films prepared with H-ALG or L-ALG. A similar release profile was obtained when each film incorporated 15 mg of LD. All films prepared with 4% PUL, 8% PUL or 3% L-ALG immediately released the water soluble drug with dissolution of the forms.

**Figure 4:**
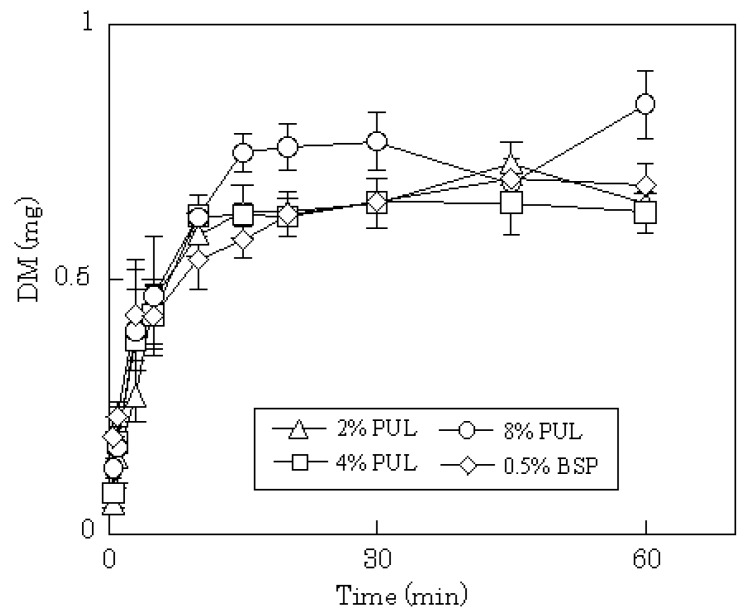
Release profiles of DM from FDFs in physiological saline.

**Figure 5:**
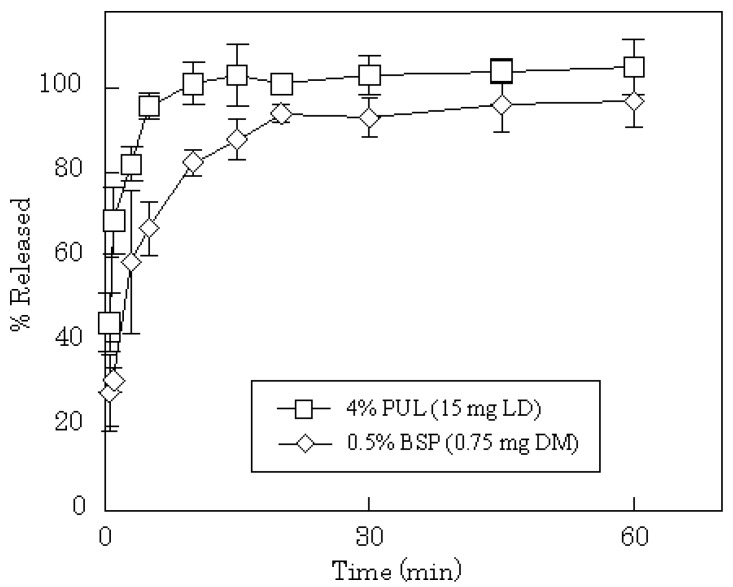
Release profiles of LD and DM from FDFs in phosphate-buffered saline (pH 7.4).

**Figure 6:**
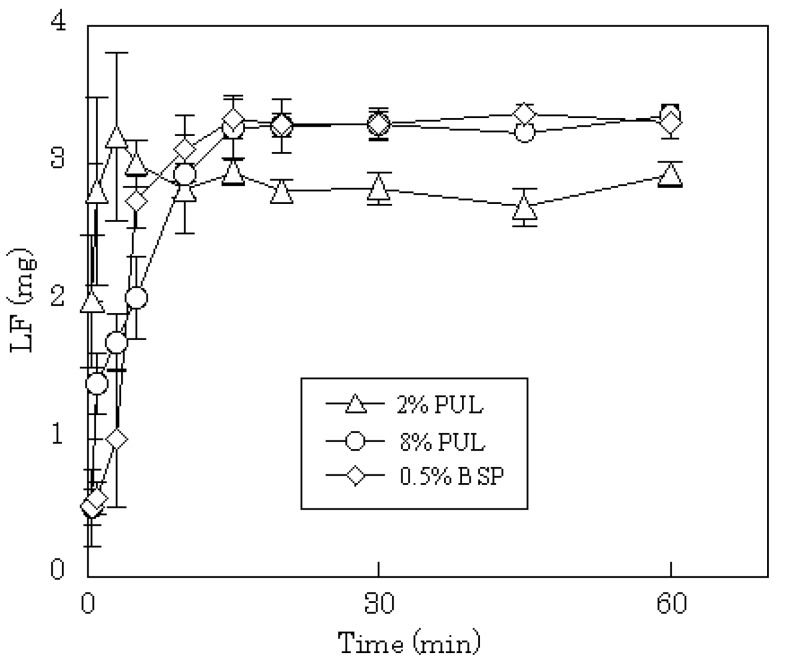
Release profiles of LF from FDFs in physiological saline.

DM release profiles from films prepared with 2–8% PUL or 0.5% BSP are shown in Figure 4. In all cases, the release of DM was complete after 15 min, although the release rate was slightly slower compared to PC or LD. In general, the dissolution rate of a poorly water-soluble drug, such as DM, into gastrointestinal fluid is affected by the particle size of the drug, which governs the surface area of the solid mass. In FDFs, the drug is dispersed as micro-particles in the water-soluble polysaccharide film matrix. Therefore, the particles of DM may dissolve simultaneously with the dissolution of the film in saliva. The anticipated release profiles were obtained in phosphate-buffered saline (pH 7.4) as shown in Figure 5. A polymer compound, LF, was released immediately from the dosage form; the initial release rate of LF from 8% PUL film decreased compared with 2% PUL as shown in Figure 6. This result might be attributed to a difference in the erosion of the matrix because it was observed with the naked eye that the dissolution rate of 8% PUL film in physiological saline was slower than that of 2% PUL film. 

## 3. Experimental Section

### 3.1. Materials

DM, PC and LF (bovine milk) were obtained from Wako Pure Chemical (Osaka, Japan), and LD was obtained from Sigma (St Louis, MO, USA). H-ALG was obtained from Kimitsu Chemical Industries (Tokyo, Japan), and L-ALG was obtained from Johnson Matthey (MA, USA). CHS (C) sodium salt was obtained Wako. PUL was supplied by Hayashibara Biochemical Laboratories (Okayama, Japan) and BSP was supplied by Morishita Jintan (Osaka, Japan). Phosphate-buffered saline (pH 7.4) was purchased from Wako. All other chemicals were of reagent grade.

### 3.2. Preparation of Polysaccharide Film

Polysaccharide solution was prepared with deionized water and the viscosity was measured with a viscometer (VM-1G-M, CBC Materials, Tokyo) at 20 °C. A model drug was added to the polysaccharide solution and mixed well, then 3.0 g of the solution was poured into a plastic Petri dish (diameter 54 mm). After 24 h at 37 °C, the film formed on the dish was stored in a dessicator. Film formation was judged not to have occurred if a film could not be removed from the bottom of the dish.

### 3.3. Film Thickness and Surface Structure

Film thickness was measured at 10 points on each film using a micrometer (CLM1–15QM, Mitutoyo, Kawasaki, Japan) with a set pressure of 0.5 N. Measurements were made on three films and the mean thickness was calculated. The shape of the film surface was observed using a laser auto-focus three-dimensional profilemeter (MAP-3D, COMS, Amagasaki, Japan).

### 3.4. Drug Dissolution test

Physiological saline or phosphate-buffered saline (pH 7.4) was used as the dissolution test medium. A film was placed in a plastic dish and 10 mL of dissolution medium incubated at 37 °C was added. The dish was shaken at 300 rpm in a shaker incubator at 37 °C. In the case of DM, LD or PC, an 80 μL aliquot was removed periodically, placed in a micro test tube (1.5 mL) and 720 μL of methanol was added to precipitate the polysaccharide dissolved from the dosage form. The sample was mixed and centrifuged (10,000 rpm, 5 min), then the supernatant was injected into an HPLC column. In the case of LF, a 25-μL aliquot was removed periodically and placed in a 96-well microplate. The amount of LF released was measured using the BCA protein assay kit (Thermo Scientific, Rockford, IL, USA) in a multi plate reader (Viento, Dainippon Seiyaku, Suita, Japan). All tests were performed in triplicate.

HPLC conditions: the HPLC system consisted of a LC-6A pump (Shimadzu, Kyoto, Japan) operating at a flow rate of 0.8 mL/min, a packed column (150 mm × 4.6 mm, Cosmosil 5C_18_-MS-II, Nacalai Tesque, Kyoto), and a SPD-6A UV detector (Shimadzu). The detector wavelength was 220 nm. Quantification of DM was conducted at ambient temperature using an eluent consisting of 10 mM sodium phosphate buffer (pH 2.3) and acetonitrile (7:3) [16]. Eluent comprising 50 mM phosphate buffer (pH 3.0) and acetonitrile (85:15) was used for quantifying LD [17]. And an eluent comprising 1.15% phosphoric acid containing 0.3% triethylamine and methanol (98:2) was used for quantifying PC [18].

## 4. Conclusions

In this study, easy-to-swell films were prepared with natural polysaccharides. The thicknesses and surface shapes were affected by the concentration of the material used for film formation. Some films were able to incorporate model compounds such as PC, LD, DM and LF. All of the films could disintegrate quickly in physiological saline. FDFs are useful dosage forms, especially for treating localized infections in the oral cavity. FDFs may be a useful dosage form for patients with diseases causing hyposalivation, such as Sjogren’s syndrome, due to their high solubility even in a restricted amount of saliva. New films comprising polysaccharide blends to control film flexibility are now being prepared. 
